# Evaluating the Burden of Lymphedema Due to Lymphatic Filariasis in 2005 in Khurda District, Odisha State, India

**DOI:** 10.1371/journal.pntd.0004917

**Published:** 2016-08-22

**Authors:** Victoria Walsh, Kristen Little, Ryan Wiegand, Jonathan Rout, LeAnne M. Fox

**Affiliations:** 1 Parasitic Diseases Branch, Division of Parasitic Diseases and Malaria, Center for Global Health, Centers for Disease Control and Prevention, Atlanta, Georgia, United States of America; 2 Church’s Auxiliary for Social Action, Odisha, India; University of Ghana, GHANA

## Abstract

**Background:**

Over 1.1 billion people worldwide are at risk for lymphatic filariasis (LF), and the global burden of LF-associated lymphedema is estimated at 16 million affected people, yet country-specific estimates are poor.

**Methodology/Principal Findings:**

A house-to-house morbidity census was conducted to assess the burden and severity of lymphedema in a population of 1,298,576 persons living in the LF-endemic district of Khurda in Odisha State, India. The burden of lymphedema in Khurda is widespread geographically, and 1.3% (17,036) of the total population report lymphedema. 51.3% of the patients reporting lymphedema were female, mean age 49.4 years (1–99). Early lymphedema (Dreyer stages 1 & 2) was reported in two-thirds of the patients. Poisson regression analysis was conducted in order to determine risk factors for advanced lymphedema (Dreyer stages 4–7). Increasing age was significantly associated with advanced lymphedema, and persons 70 years and older had a prevalence three times greater than individuals ages 15–29 (aPR: 3.21, 95% CI 2.45, 4.21). The number of adenolymphangitis (ADL) episodes reported in the previous year was also significantly associated with advanced lymphedema (aPR 4.65, 95% CI 2.97–7.30). This analysis is one of the first to look at potential risk factors for advanced lymphedema using morbidity census data from an entire district in Odisha State, India.

**Significance:**

These data highlight the magnitude of lymphedema in LF-endemic areas and emphasize the need to develop robust estimates of numbers of individuals with lymphedema in order to identify the extent of lymphedema management services needed in these regions.

## Author Summary

The results presented in this article demonstrate the need to assess the clinical disease burden due to lymphatic filariasis (LF) in areas of the world where this disease occurs. There are effective strategies that can be implemented to reduce the suffering and morbidity associated with chronic filarial infections. These include lymphedema management programs that teach basic hygiene to decrease limb infections that contribute to lymphedema progression. These interventions should be implemented on a broader scale and can be integrated with other chronic disease prevention programs at subnational and national levels. However, knowledge of the scale and distribution of individuals suffering with lymphedema in countries with LF is required to decide where and how these health services should be implemented. Countries with LF are encouraged to conduct burden assessments to decide how best to implement lymphedema management programs. Providing health services for those with lymphedema is an important component of reducing LF as a public health problem.

## Introduction

**Fig 1:**
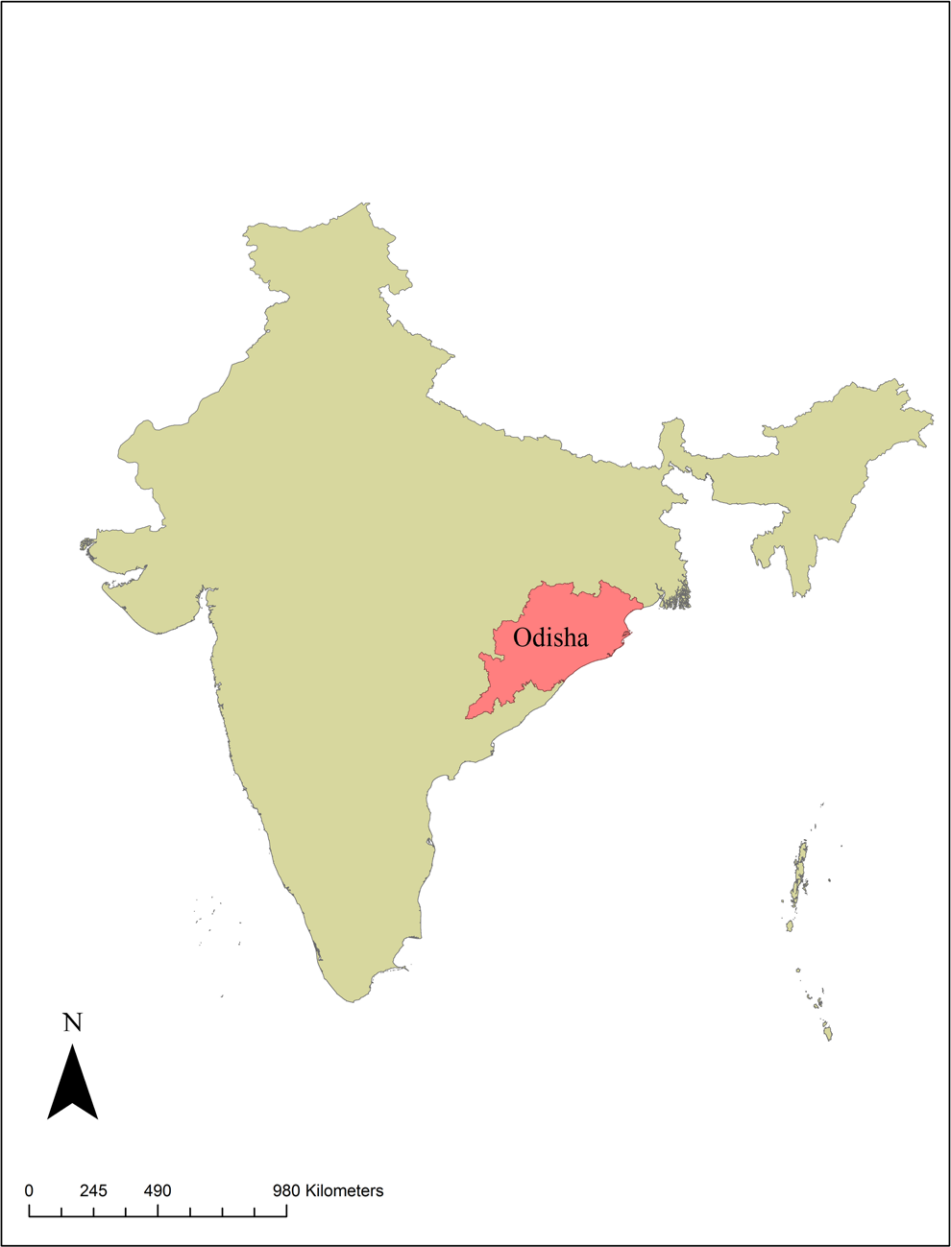
Map of India, highlighting Odisha State. (Map generated in ArcGIS 10.2).

Lymphatic filariasis (LF) is a mosquito-borne parasitic infection that damages the lymphatic system and can cause chronic and debilitating swelling of the limbs known as lymphedema or, in its more advanced form, elephantiasis. Approximately 1.1 billion people are at risk for LF in more than 73 countries worldwide [1], 600 million of whom reside in 250 districts in India accounting for over 40% of the global LF burden [2]. An estimated 50% of India’s LF endemic population resides in northeastern India in areas of poverty [3–5]. Among the LF endemic regions, Odisha state (Fig 1), one of the poorest states in India, was recognized historically as one of the most highly endemic and remains as such [4, 6, 7].

Similar to the World Health Organization’s (WHO) global goal to eliminate LF as a public health problem by 2020, India has set a national goal of LF elimination which focuses on two pillars: 1) interruption of disease transmission through mass drug administration (MDA) and 2) reduction of LF-associated morbidity for patients already impacted by clinical disease. MDA campaigns have been occurring in India since 1997 and have progressed well with some areas having demonstrated interruption of transmission [3, 8, 9]. However, it is recognized that MDA does not treat the chronic clinical manifestations, specifically lymphedema, elephantiasis and hydrocele, seen in some LF-infected individuals [2, 10]. Therefore, for individuals with lymphedema and elephantiasis, lymphedema management programs are necessary to mitigate the symptoms associated with lymphedema, and to prevent the development of episodes of adenolymphangitis (ADL) known to lead to the worsening of lymphedema [11, 12]. Accurate estimates of lymphedema and hydrocele patients are needed by national LF elimination programs to inform service provision, yet robust assessments have been rarely performed [10, 13]. An emphasis needs to be placed on obtaining accurate patient estimates in order to properly scale up morbidity management and disability prevention services for LF patients with clinical disease. Enumeration of individuals with early lymphedema (Dreyer stages 1 and 2) is particularly valuable as these individuals have the potential to gain the most benefit, compared to those with later stages of lymphedema, from lymphedema management programs [14]. Although outside the scope of this survey, estimates of number of patients with hydrocele are also necessary for country programs to ensure that sufficient surgical resources are available for these individuals. The objective of this analysis was to describe and analyze data obtained from a thorough method of enumerating individuals with lower limb swelling in a highly-LF endemic district in Odisha State, India.

## Methods

### Ethics Statement

Permission for the morbidity census was obtained from the Odisha State Department of Health and Family Welfare. The morbidity census was determined to be program evaluation under CDC policy.

### Census Area and Design

**Fig 2:**
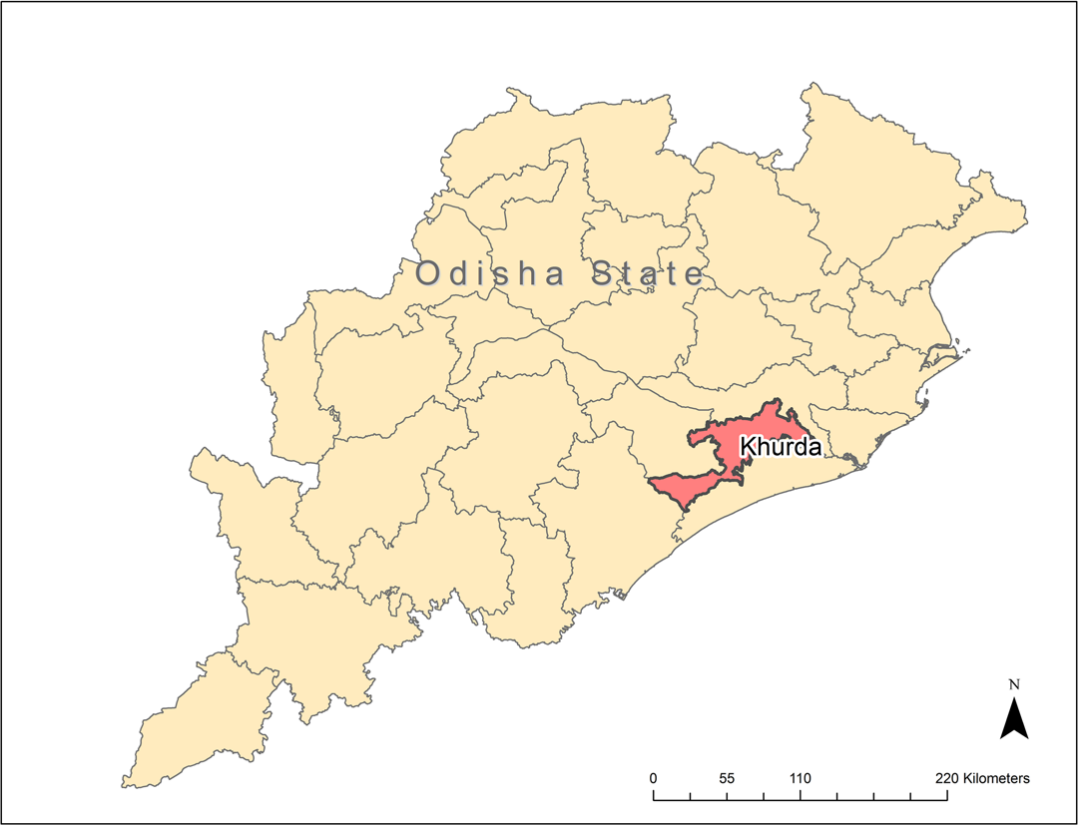
Khurda District, Odisha State, India. (Map generated in ArcGIS 10.2).

The morbidity census took place in rural and peri-urban areas of Khurda district in Odisha state (Fig 2) over a four month period in 2005 (approximate population 1.3 million). All individuals living in Khurda district yet outside the capital city of Bhubaneswar were included in the morbidity census. Prior to this census it was well established that Khurda was highly endemic for Bancroftian filariasis; however, more robust estimates of individuals with lymphedema were needed in preparation for the initiation of a district-wide community-based lymphedema management program [6, 8, 11, 12, 15]. In 2005, a local non-governmental organization (NGO), Church’s Auxiliary for Social Action (CASA) in partnership with approximately forty other local NGO’s working in Khurda district, conducted a house-to-house morbidity census to enumerate the number of individuals with lymphedema/elephantiasis in the district. Approximately 200 community health workers and NGO volunteers were trained by government workers and other filarial experts in patient enumeration and clinical diagnosis in accordance with the Dreyer manual on basic lymphedema management and the Indian government’s manual for *Operational Guidelines on Elimination of Lymphatic Filariasis* [16, 17]. Staging was not validated for all the patients enumerated, but a small sample of patients did undergo validation of staging as part of an evaluation of the program. The details on these patients are discussed in the following publications [16, 17]. Trained staff documented persons reporting lymphedema in each household, and staged the degree of lymphedema according to the Dreyer staging system [16]. Communication with individuals occurred in the local language, Oriya. Standardized questionnaires were administered by trained staff and data on basic demographics and risk factors for lymphedema were collected for all lymphedema patients. Additionally, trained staff asked individuals with lymphedema to report the occurrence and number of ADL episodes they may have experienced at any time during the last twelve months. We recognize that recalling the number of ADL episodes over one year may be difficult for patients, particularly those who have had numerous ADL episodes and subsequent work we have done would recommend asking about ADL episodes over a shorter period of time, such as one month [11, 12]. Data on individuals reporting lymphedema were recorded on paper registers by NGO volunteers. Over a period of two months following completion of the morbidity census, data were double data entered into Microsoft Excel by CASA data management staff.

### Morbidity Census Questionnaire

The morbidity census enumerated the number of individuals with lymphedema of the leg in Khurda district by village. All households in the district were asked about the presence of individuals with lymphedema in the household. Data collected on individuals with lymphedema included gender, age, caste, religion, level of education, village of residence, rural/peri-urban residence, presence of leg swelling, lymphedema stage (Dreyer scale), number of ADL episodes in the past year, and presence of another family member with lymphedema.

### Definitions

For the purposes of this paper, lymphedema refers to swelling observed in the lower limbs—right or left leg, as this is the most commonly observed location for filarial-associated lymphedema. Lymphedema severity was established based on the seven stage classification system developed by Dreyer, et al. [16]. ADL episodes were defined as any period of pain, redness, and/or swelling of the affected leg, which may have been accompanied by fever and/or chills [18, 19].

### Data Analysis

#### Mapping

The geographic distribution of lymphedema in Khurda district, Odisha State, India was described by mapping the village-level prevalence. Maps were created in ArcGIS 10.2 (ESRI, Redlands, California) and shapefiles for Odisha State were obtained from Geographic Enterprises, LLC (Wake Forest, NC). The 982 unique villages reporting one or more lymphedema patients were matched to the locations in ArcGIS using Microsoft Excel (Microsoft Corp., Redmond, VA) at the village and block levels using the Khurda district shapefile containing data from the 2001 Indian census. Village-level prevalence was calculated by dividing the counts of individuals reporting lymphedema by the projected 2005 village population, extrapolated from the 2001 and 2011 Indian censuses [20]. Total prevalence was calculated including all individuals reporting lymphedema. Counts for early stage lymphedema included Dreyer stages 1 and 2.

#### Logistic regression

Regression analysis was restricted to individuals over the age of 15 years who reported lymphedema since filarial lymphedema generally begins post-puberty. Poisson regression was used for patient counts, allowing interpretation of the effects in terms of prevalence ratios (PR) and adjusted prevalence ratios (aPR). Generalized Estimating Equations (GEE) were used in all modeling to account for correlations at the village level [21]. An exchangeable correlation structure was utilized. All data were analyzed in SAS version 9.3 (SAS Institute, Inc., Cary, North Carolina).

## Results

### Morbidity Mapping

**Fig 3:**
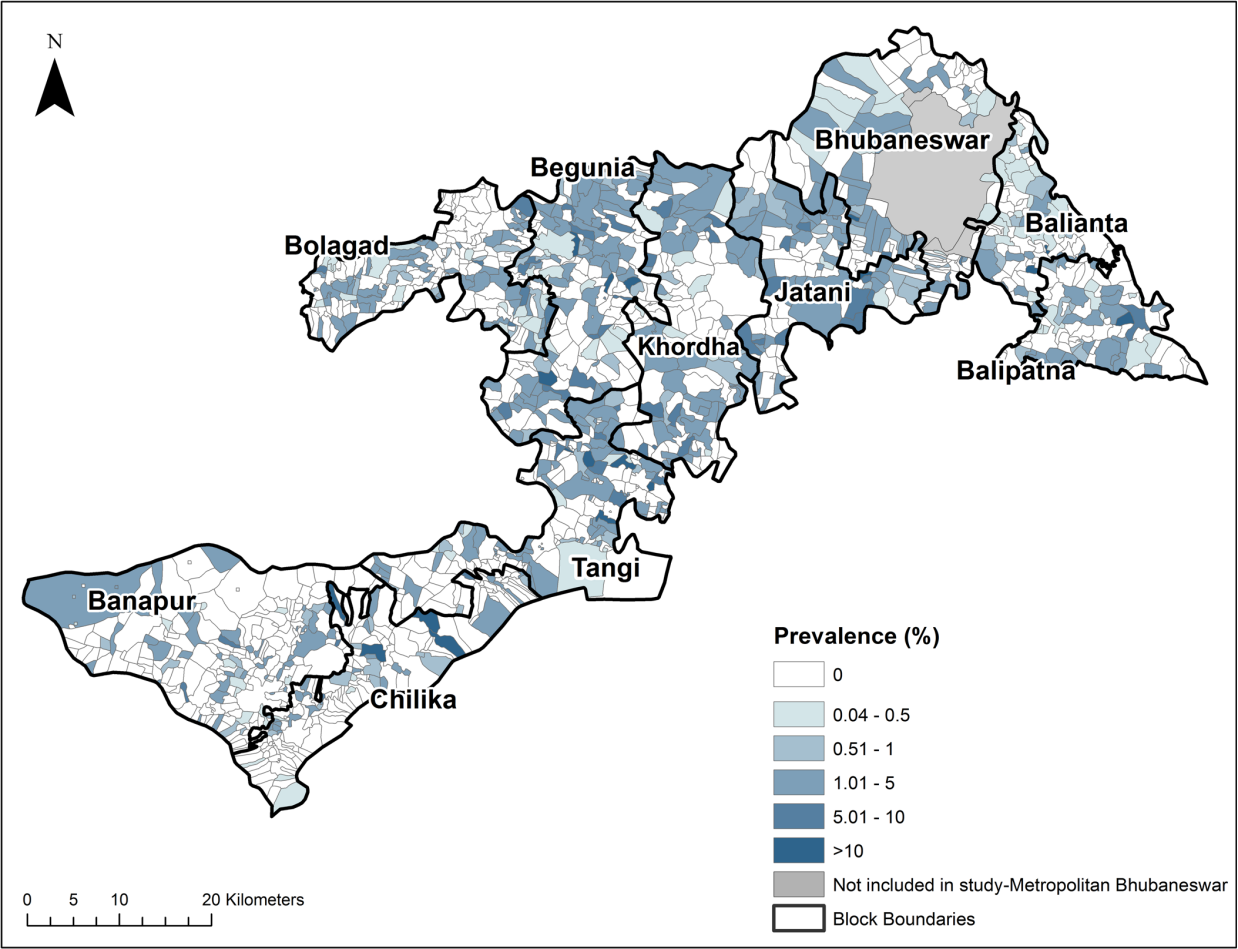
Village-level prevalence of lymphedema in Khurda District, Odisha State, India. (Map generated in ArcGIS 10.2).

**Fig 4:**
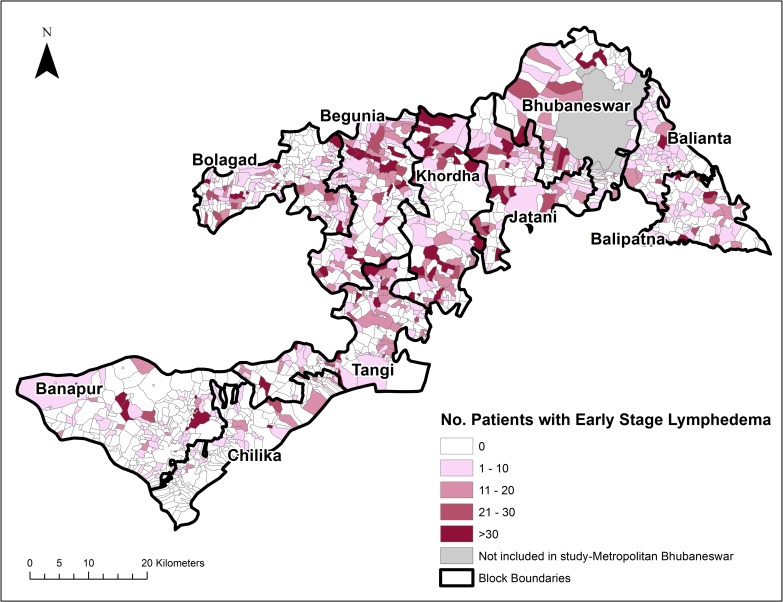
Early stage lymphedema patients by village in Khurda District, Odisha State, India. (Map generated in ArcGIS 10.2).

Approximately 68% of the villages listed in the CASA census were correlated to the Indian census, the remaining 32% either used smaller naming references than the Indian census (such as hamlet or ward) or were referred to by a different name at the time of the CASA census. Fig 3 illustrates that persons with lymphedema are present in over 50% of villages in Khurda district. Furthermore, Fig 4 shows the distribution of individuals with early lymphedema (stage 1 and stage 2) by village throughout the district. Mapping of lymphedema prevalence demonstrates the widespread burden of this chronic condition throughout Khurda district. The geographic distribution of individuals with lymphedema including those with early stage (Dreyer stages 1 & 2) is widespread throughout the district.

### Demographics

Data on 17,036 individuals reporting lymphedema were recorded. 51.3% of the individuals were female, mean age 49.4 years (Table 1). Two-thirds (67%) of individuals reported early lymphedema (Dreyer stages 1 & 2), 22% reported moderate lymphedema (Dreyer stage 3), and 10% reported advanced lymphedema (Dreyer stages 4–7). Mean lymphedema stage was 2.1 (range 1–7). The majority of the study population (83.9%) reported a history of ADL episodes; mean number of ADL episodes per year was 1.05 (range 0–3). Additionally, 21.5% of the individuals reported family members with lymphedema as well.

**Table 1:** Morbidity census demographics, Khurda District, Odisha State, India (N = 17,036).

Characteristic	N	%/ Range
**Female gender**	8,746	51.3%
**Mean age (years)**	49.40	3–96
**Lymphedema Stage**		
Early (Stages 1 & 2)	11,484	67.4%
Moderate (Stage 3)	3,794	22.3%
Advanced (Stages 4–7)	1,758	10.3%
**Mean lymphedema stage**	2.10	1–7
**History of ADL episodes**	14,284	83.9%
**Mean number of ADL episodes per year**	1.05	0–3
**Family members with lymphedema**	3,670	21.5%

### Risk Factors for Advanced Lymphedema

In univariate analysis, the prevalence of lymphedema among females was less than the prevalence of lymphedema among males (PR: 0.84, 95% CI 0.77, 0.93) (Table 2). Additionally, there was a significantly higher prevalence of advanced lymphedema with increasing age (p-value <0.0001). When compared with persons aged 15–29 years, persons 70 years and older had more than a three-fold higher prevalence of lymphedema (PR: 3.47, 95% CI 2.56, 4.71). Individuals who reported two ADL episodes in the previous year were more than five times as likely as individuals who reported no ADL episodes in the previous year to have advanced lymphedema. Interestingly, individuals who reported having a family member with lymphedema had on average a 30% greater prevalence of advanced lymphedema compared with persons who did not report a family member with lymphedema (PR: 1.30, 95% CI 1.12, 1.49). The results from the multivariate analysis support the associations observed in univariate analysis (Table 2).

**Table 2:** Risk factors for advanced lymphedema (Stages 4–7) in surveyed individuals ages 15–99 in Khurda District, Odisha State, India (N = 16,909).

Variable	Unadjusted Estimates			Adjusted Estimates		
	PR	95% CI	P-Value	aPR	95% CI	P-Value
Female gender	0.84	(0.77, 0.93)	0.0004	0.87	(0.80, 0.95)	0.0020
Advancing age						
Age group (15–29)	Ref	Ref		Ref	Ref	
Age group (30–39)	1.35	(0.97, 1.87)	0.074	1.33	(1.00, 1.78)	0.0524
Age group (40–49)	1.99	(1.46, 2.72)	< .0001	1.94	(1.48, 2.55)	< .0001
Age group (50–59)	2.54	(1.88, 3.44)	< .0001	2.50	(1.89, 3.23)	< .0001
Age group (60–69)	3.17	(2.33, 4.31)	< .0001	3.00	(2.29, 3.93)	< .0001
Age group (70 and over)	3.47	(2.56, 4.71)	< .0001	3.21	(2.45, 4.21)	< .0001
ADL episodes within the past year						
0 ADL episodes	Ref	Ref		Ref	Ref	
1 ADL episode	1.25	(1.03, 1.51)	0.0215	1.15	(0.97, 1.38)	0.1136
2 ADL episodes	5.72	(3.60, 9.10)	< .0001	4.65	(2.97, 7.30)	< .0001
3 ADL episodes	2.77	(2.15, 3.58)	< .0001	2.51	(1.96, 3.22)	< .0001
Family members with lymphedema	1.30	(1.12, 1.49)	0.0004	1.25	(1.09, 1.43)	0.0010

## Discussion

This house-to-house morbidity census provides an example of a comprehensive method for estimation of individuals with lymphedema in a LF-endemic region. Conducted at the district level in India, with a population of more than 1.2 million people, this census demonstrates that enumeration of individuals with lymphedema is possible on a large scale. Our findings suggest that the prevalence of lymphedema in Odisha, India in 2005 was considerable, affecting 1.3% of the population. Previously, the prevalence of lymphedema was estimated to affect 3.4% of the population, while 2014 burden assessments estimate the number of lymphedema patients in Odisha State to be approximately 80,000 [15, 22]. While an MDA campaign had occurred in Odisha in 1997, consistent annual MDA in Odisha did not begin until 2004. Therefore the prevalence of lymphedema calculated from the results of the morbidity census would not have been significantly impacted by MDA.

Conducting the morbidity census as a house-to-house survey allowed for the creation of maps detailing the distribution and prevalence of lymphedema at the village level, which had not previously been achieved. The greatest proportion (67.5%) of participants reported early lymphedema, the burden of which is widespread throughout the district (Fig 4). The significant number of individuals with early stage lymphedema highlights the large percent of the population that may benefit from the establishment of lymphedema management programs that target modifiable disease risk factors such as ADL episodes [1, 13, 23, 24]. Identifying that the prevalence of lymphedema is geographically widespread emphasizes the need for scaling up lymphedema management services and training health care workers throughout the district. Furthermore, the results of this morbidity census informed communities of the burden of lymphedema in their villages and identified the need for lymphedema management resources in individual communities. The census was necessary in order to determine the number of human and financial resources needed to implement the community-based lymphedema management program in Khurda, specifically the number of NGO volunteers, commodities for lymphedema management, training materials, etc. Based on prior experiences with lymphedema management programs, CASA determined that one NGO volunteer was needed to train and follow-up with twenty lymphedema patients.

In addition to providing mass drug administration (MDA) to prevent LF infection, the Indian national lymphatic filariasis elimination program has implemented an LF morbidity management and disability prevention program that involves training of health care professionals and of community health workers (P.K. Srivastava, personal communication). The establishment of community-based lymphedema management programs in Odisha State has demonstrated success in the following areas: prevention of ADL episodes, halting lymphedema progression, improving quality of life, improving patient perceptions of their disability, and improving work productivity for lymphedema patients living in this region [11, 12]. The efforts of partnering NGOs and the response of the Indian national LF elimination program can serve as an example to other LF elimination programs in scaling up their morbidity management and disability prevention (MMDP) efforts. We recognize that a house-to-house morbidity census for LF-related disease may not be feasible for all LF-endemic districts given human and fiscal resource constraints. Other approaches to morbidity assessments have involved the use a community-led standard messaging system (SMS) reporting tools piloted in Malawi and Ghana, as well as a 30 cluster survey that was conducted to estimate the lymphedema and hydrocele burden in Bangladesh [25, 26]. We recommend that further operational research be performed to develop more cost-effective tools for assessment of LF-related disease burden to inform countries preparing to scale up MMDP services.

Analysis of the risk factors for advanced lymphedema provided further evidence to support the need for lymphedema management programs in this region. Among individuals aged 15 and older who reported lymphedema, older age (70 years and older) was highly associated with an increased prevalence of advanced stage lymphedema; which is consistent with findings in other LF endemic areas, such as Haiti [24]. The increase in prevalence of lymphedema with increasing age corresponds to the fact that lymphedema symptoms often do not appear until years or even decades after an individual is first infected and they tend to worsen over time and with the number of ADL episodes a person experiences. In addition, this in-depth morbidity census confirmed the observation reported previously in smaller studies that advanced lymphedema is associated with the number of ADL episodes reported during the previous year [11–13]. These findings emphasize the opportunity for prevention of lymphedema progression and possibly disability through implementation of programs that reduce the severity and frequency of ADL episodes in LF-endemic regions.

Quantifying the number of individuals with lymphedema and hydrocele through morbidity censuses such as this one can be used to calculate the economic impact of the disability associated with the chronic manifestations of LF infection. Numerous studies have evaluated the economic impact of the chronic manifestations of LF infection for both estimated financial loss and number of work days lost [2, 27–30]. Ramaiah et al. [29, 30] provided evidence for the significant costs associated with chronic manifestations as well as ADL episodes, including an estimated $842 lost annually to families needing to fund treatments and losing work days [28]. Additionally, calculations using average hours worked per day, average hours of work-time lost, and patient estimates for India determined that 1,098 million days of work were lost due to chronic LF infection in India annually [28]. The decrease in morbidity associated with lymphedema management programs has been shown to save on average 1.7–3.5 days of labor per patient resulting in significant financial savings for families and communities, especially in highly LF-endemic areas [11, 12]. By calculating accurate estimates of those with chronic manifestations of LF infection, countries can better understand the local economic impact of LF- related disease. These data can inform interventions that effectively reduce this burden both at local and national levels as a complement to ongoing efforts focused on interruption of LF transmission.

This analysis was subject to a number of limitations. It is uncertain whether all patients identified in the census had LF-associated lymphedema or lymphedema due to another cause given that no serologic testing was performed. However, LF is the leading cause of lymphedema in Odisha, and lymphedema management programs are needed for individuals affected by lymphedema regardless of the cause. Additionally, strengthening country health systems through the integration of LF lymphedema management programs with leprosy, Buruli ulcer and podoconiosis programs has been recommended by WHO in the aide-memoire [31]. Secondly, because of the stigma associated with this chronic manifestation of LF infection, it is possible that patients were unwilling or reluctant to divulge their status to surveyors. The results of this census, then, may underestimate the true burden of lymphedema. As the staff performing the census were not trained health care professionals, they did not perform extensive physical examinations of individuals with lymphedema which may have impacted the lymphedema staging leading to an overestimate of stage 1 patients given that stage 1 corresponds to any minor swelling that may not have been LF-related. Additionally, in this census there is the potential for recall bias when patients were asked to report the number of ADL episodes they experienced in the last year which may have been an underestimate given other publications that have reported higher numbers of ADL episodes within 30 day to 6 month time frames [24, 26]. Lastly, the morbidity census and Indian census data were reported at different levels of region names. This explains why only 67% of the morbidity census data was able to be mapped, the remaining data included levels of residence that did not directly match the Indian census and due to the limitations of the time since the morbidity census occurred it was not feasible to reconcile these discordant villages.

MDA campaigns have demonstrated great success in interrupting the transmission of LF, and as we continue to move forward towards the goal of elimination of LF as a public health problem countries will need to ensure morbidity management services are available for those patients already experiencing chronic sequelae associated with LF infection. In most LF-endemic countries the burden of filarial disease is poorly defined and is often underestimated. The data obtained from a morbidity census can provide program managers with a sense of the widespread geographic burden of disease. The methodology of this census can serve as an example for other LF-endemic countries seeking to enumerate lymphedema patients at the district and national level to meet the dossier requirements for morbidity management and disability prevention set by WHO for LF elimination. Scaling-up morbidity management programs is an essential component of global LF elimination as it was the recognition of the suffering of patients with chronic infection that inspired setting of the goal of global LF elimination in the first place.

## Supporting Information

S1 Checklist STROBE checklist. (DOC)
